# Recognizing the preventive quality in the adoption of innovations: The case of third-party ownership photovoltaic systems in Finland

**DOI:** 10.1016/j.heliyon.2023.e21907

**Published:** 2023-11-12

**Authors:** Deborah Kuperstein-Blasco, Saku Mäkinen

**Affiliations:** a Industrial Engineering and Management, Tampere University, Korkeakoulunkatu 7, 33720, Tampere, Finland; b Department of Mechanical and Materials Engineering, University of Turku, 20500 Turku, Finland

**Keywords:** Photovoltaic systems, Third-party ownership, Preventive quality, Adoption of innovations, Diffusion of innovations theory, PLS-SEM

## Abstract

This study explores the role of the preventive quality of innovations on their adoption intention. The preventive quality of innovations is a distinctive feature of innovations that is directed towards avoiding a future, possibly harmful event. Empirically grounded in third-party ownership of photovoltaic systems in Finland, this study examines data collected from an online survey measuring respondent intention to adopt. A series of hypotheses theoretically grounded in the Diffusion of Innovations theory and the preventive quality of innovations were tested through Partial Least Squares Structural Equation Modeling with SmartPLS4. Findings for the overall sample reveal that the preventive quality and the relative advantage of innovations influenced adoption intention positively. Findings highlight the preventive quality of innovations as a construct that acts as the greatest contributor to the dependent variable.

## 1 Introduction

The study of innovation adoption has seldom considered the preventive quality of innovations outside of health-related applications. The preventive quality of innovations is a distinctive feature of innovations that is directed towards avoiding a future, possibly harmful event. The *preventive quality* label does not categorize the innovation but describes a main or underlying feature of the innovation concerning prevention. Among the innovations that can be given the preventive quality label, there are information security behaviors preventing cyber-attacks [1] wearable devices preventing future diseases [2], green innovations and behaviors preventing environmental damage [3,4] and the use of wooden materials preventing poor indoor air quality in buildings [5].

While prevention represents possibilities to avoid or mitigate future unwanted events, it also conveys difficulties, as preventive efforts are difficult to foster [6]. Two important features of prevention illustrate why it is so difficult to promote. First, we need to know the cause of what we are trying to prevent, second, prevention is to be viewed from a long-term perspective [6]. This conflictive relationship between prevention and future-oriented benefits can challenge the adoption of innovations with preventive qualities.

In this paper, we pay attention to the preventive quality of innovations on their adoption intention, our objective is to conceptualize the preventive quality as a construct of innovations and identify how the preventive quality of innovations influences adoption intention. The study is empirically grounded in the adoption intention of residential photovoltaic (PV) systems through third-party ownership (TPO) seeking to identify the role of the preventive quality of PV systems. Here, we study the preventive quality of PV systems regarding climate change prevention and mitigation as these help lower GHG emissions [7]. We carry out our study through a series of hypotheses grounded in the preventive quality of innovations and Diffusion of Innovations theory through Partial Least Squares Structural Equation Modeling.

The adoption of residential PV systems has been studied from multiple perspectives based on the diffusion of innovations (DOI) theory, behavioral theories including the Technology Acceptance Model, TAM [8], the Theory of Planned Behavior [9], and the Unified Theory of Acceptance and Use of Technology, UTAUT [10], peer effects [11,12], socioeconomic factors [13,14], environmental and knowledge factors [15], and patterns of social acceptance [16], among others. Various publications focus on factors driving adoption across countries including the United States [7], Japan [17], and Mexico [13] as well as Northern locations such as Norway [18] and Finland [19,20]. Studies carried out in cross-national settings [21,22] highlight that adoption intention is context-specific and not easily generalizable across contexts.

The role of prevention has not been covered by behavioral theories that are frequently utilized to study the acceptance of technologies, such as the Technology Acceptance Model, TAM [23], the Theory of Planned Behavior [24], or the Unified Theory of Acceptance and Use of Technology, UTAUT [25]. Prevention has been covered by the Protection Motivation Theory (PMT) [26] in health-related applications through the constructs of threat appraisal and coping appraisal. These constructs have been modified in previous studies to understand behavioral adaptations in situations involving risk [2,4]; however, none of these constructs reveal the influence of the preventive quality of the innovation per se. Through this study, we contribute to findings from less obvious solar-producing locations [18,19] by exploring the perspective of prevention through DOI theory, seeking to build future research avenues that incorporate more frequently utilized behavioral theories.

Successes of prevention have come primarily as a result of economic and social pressure [27,28]; contemporary sustainability issues and objectives to build resilient societies [29] could create the necessary pressure that influences the adoption of innovations with preventive qualities. This could contribute to creating resilience capacities that aid systems, institutions, and people to cope with, withstand, and bounce back from shocks [29].

The rest of the paper is divided into five sections. Section 2 presents the theoretical framework of prevention and attributes of innovations to build hypothesized relationships. Section 3 presents survey measures and introduces Partial Least Squares Structural Equation Modeling as the methodology for statistical analysis and testing the framework. Section 4 presents the results of the empirical analysis. Finally, section 5 provides discussions, summarizes study conclusions, and identifies limitations and areas for future work.

## 2 Theory background and hypothesis development

### 2.1 Prevention and the adoption of innovations

Efforts to study the adoption of innovations considering their preventive quality and the benefits incurred from prevention have been mostly directed toward health innovations. For example, D'Souza et al. [30], Adams et al. [31], and Head and Cohen [32] studied the adoption of the Human Papillomavirus (HPV) vaccine by focusing on prevention, highlighting reasons underlying diffusion. Similar studies have been directed toward innovations that prevent other ailments such as HIV/AIDS [33] and smoking and respiratory diseases [34].

There is a multitude of innovations that seek to prevent an unwanted event and whose adoption could be studied considering the role of their preventive quality. However, only a handful of these innovations has been studied considering their preventive quality, these include information security behaviors preventing cyber-attacks [1], wearable devices preventing future diseases [2], green innovations and behaviors preventing environmental damage [3,35], and the use of wooden materials preventing poor indoor air quality in buildings [5]. There is a deficit of studies exploring the role that the preventive quality of innovations plays over adoption intention.

The role of prevention has been partially covered by the Protection Motivation Theory (PMT) [26], regarded as a more comprehensive extension of the Health Belief Model (HBM), in the constructs of threat appraisal and coping appraisal; however, these constructs seek to explain individual perception and response to a situation involving risk and do not study the preventive quality of the innovation per se. Thus, there are still opportunities to explore the preventive quality of innovations as an independent construct contributing to adoption intention.

As studies examining the preventive quality of innovations have not explored its role in adoption intention, we refer to the literature on preventive behavior and prevention processes to hypothesize this relationship. Prevention is regarded as a difficult and slow process. The outcome of successful prevention is a non-event, and a definite ‘now’ is not likely to be exchanged for an uncertain ‘then’ [6]. Prevention of an unwanted event varies from simple behaviors that require a one-time decision, to complex behavioral changes that must be sustained [36]. Prevention becomes more difficult to foster when behavioral changes must be repeated [37,38] and preventive efforts are frequently lost due to follow-up [39]. In some cases, preventive efforts are counterproductive compared to their non-preventive counterpart, as in public policy, where voters reward politicians who pursue relief spending rather than prevention spending [40]. Considering these attributes of prevention, we propose that.

(H1) The preventive quality of innovations has a negative effect on adoption intention

### 2.2 Attributes of innovations as factors of diffusion

The Diffusion of Innovations (DOI) theory as derived by Ryan and Gross [41] and classified by Rogers [42] explains how innovations are adopted by individuals; this theory has been extensively utilized across disciplines due to its highly predictive nature. According to DOI theory, innovations' attributes help explain their different adoption rates. The attributes of innovations have been used in previous studies as predictors in the adoption of innovations centered around prevention [30,33,43,44]; these are relative advantage, compatibility, trialability, observability, and complexity. There is a large body of evidence highlighting the influence of these five attributes in the adoption of innovations. As highlighted previously, the adoption intention of PV systems is context-specific and not easily generalizable across national contexts. Therefore, to hypothesize the relationships between the attributes of innovations and the adoption intention of PV systems we will rely on previous empirical studies in PV systems and similar technologies.

Relative advantage is the degree to which an innovation is considered better than previous ideas [42, p. 15] and can be measured in economic terms, social prestige, convenience, and satisfaction factors. Traditionally this attribute is considered the strongest predictor in the adoption of innovations; the greater the perceived relative advantage of an innovation, the faster it will be adopted. Potential adopters often seek to decrease uncertainty associated with the innovation by understanding its relative advantage [45].

Empirical studies point to the influence of relative advantage in the adoption of innovations with preventive qualities, such as PV systems. In the study of PV systems, Wolske [7], Alam et al. [8], and Powell et al. [46] found relative advantage to have the strongest effect over adoption intention and adoption rates. In a study setting evaluating renewable energy technology adoption, Elmustapha et al. [47], found relative advantage to be among the top three innovation characteristics influencing adopter behavior. Similarly, Phillips and Lindquist [48] found innovators to assess relative advantage as one of the most important innovation characteristics in the adoption of green infrastructure innovations. Across these cases, relative advantage was present through elements such as novelty seeking [7], cost-effectiveness [46,48], and convenience [47].

Based on the empirical evidence on the positive and significant effect that relative advantage has over adoption intention, we hypothesize that.

(H2) The relative advantage of an innovation has a positive effect on its adoption intention.

Trialability is the degree to which an innovation can be experimented on a limited basis [42, p. 16] where ideas that can be tried on a partial basis are more likely to be adopted than those that cannot [41] as it represents less uncertainty. Potential adopters can identify if it works under their conditions by trying out an innovation. If an innovation can be easily tried, it will have a more rapid rate of adoption [42, p. 270].

Empirical evidence highlights the influence of trialability in the adoption of innovations with preventive qualities. In the study of the adoption intention of PV systems, Powell et al. [46] found trialability along with complexity to influence the time to peak adoption of solar PV, where a change in either factor would change adoption rates significantly. While PV systems have difficult trialability, they can be scaled (for example, installing solar PV in one site and proceeding to other sites). Once installed, the benefits of solar PV are realized and easily evaluated [46]. Similarly, Alam et al. [8] identified trialability as a predictor that increases the intention to adopt. Trialability was also found to be correlated with other attributes, including attitude toward the technology [7,8], beliefs [7], and observability [8,46]. Considering the role of trialability in the adoption of innovations with preventive qualities, we hypothesize that.

(H3) The trialability of an innovation has a positive effect on its adoption intention.

Observability is the degree to which the results of an innovation are visible to others [42, p. 16] where the easier it is to see the results the more likely the innovation will be adopted. While some ideas are easy to communicate and be observed by potential adopters, others are not; however, the innovation itself can be visible and stimulate peer discussion among members of a social system [12]. Peer observation and influence can promote the diffusion of innovations [11].

The observability of innovations with preventive qualities can be challenged [49] due to the long-term perspective of prevention [6]. However, the innovation itself can be visible and stimulate peer discussion among members of a social system [12], as it happened with residential photovoltaic (RPV) systems where seeing others with the RPV system influenced adoption intent. Phillips and Lindquist [48], found the observability of benefits as the least important innovation characteristic influencing willingness to adopt green infrastructure innovations. However, when discussing the characteristics of observability in interviews, respondents pointed to positive qualitative effects of the innovation's visibility, such as gaining public approval for projects that may be initially met with resistance [48]. Based on this evidence, we argue that.

(H4) The observability of an innovation has a positive effect on its adoption intention.

Compatibility is the degree to which an innovation is in line with existing values, past experiences, and needs of potential adopters. More compatible ideas will be adopted more rapidly than those not [42, p. 16]. Compatibility allows individuals to give meaning to the new idea, so it is regarded as familiar. A more compatible innovation will require a smaller behavioral change and will be adopted faster [50].

Empirical evidence points to the role of compatibility in the adoption of innovations with preventive qualities. In the study of PV adoption intention, Alam et al. [8] found that those who feel PV systems are compatible with their culture, needs, and usability are more likely to adopt these and reported it as the second most important predictor of adoption. In the context of green infrastructure innovations, Phillips and Lindquist [48] identified compatibility along with relative advantage as the two most important innovation characteristics influencing potential future adopters of green infrastructure innovations. High compatibility was also identified as a factor that explains the adoption of renewable energy technologies in the context of solar water heaters [47]. Considering this evidence, we argue that.

(H5) The compatibility of an innovation has a positive effect on its adoption intention.

Complexity is the extent to which an innovation is difficult to understand and use [42, p. 16] where ideas that are simpler to understand will be adopted more quickly; however, innovations that are too simple do not usually spread [51]. Complexity is an important barrier to the adoption of some innovations, but may not be as important as relative advantage for other innovations [47]. Complexity has been used interchangeably with the constructs of perceived ease of use [8] and effort expectancy [52] when evaluating the difficulty to use an innovation.

While innovations with preventive qualities are not necessarily more complex to understand and use than others, the cause-and-effect relationships can be complex [53]. This is illustrated in an empirical study by Vasseur and Kemp [53], who found PV systems to be complex particularly when trying to understand how these can contribute to the reduction of carbon dioxide. In their study, complexity was not found as a predictor of adoption intention, but instead as one of the reasons why adoption had not occurred. Other empirical settings point out that lower complexity leads to higher PV system adoption intention [8,46] and higher complexity lowers the likelihood of adoption [47]. Therefore, we argue that.

(H6) The complexity of an innovation has a negative effect on its adoption intention.

The role of sociodemographic factors in adoption intention has also been covered across studies in the adoption of innovations with preventive qualities. Wolske et al. [7] found a significant role of sex in the adoption intention of PV systems yet Palm [54] identified no significance of sex in solar PV adoption. Income has also been identified as a predictor in the adoption of innovations with preventive qualities where higher [55], moderate [15], and lower [7] income are correlated with adoption and adoption intention. Similarly, empirical evidence identifies household age [55] as significant in adoption intention. Hence, we propose that.

(H7) Sociodemographic factors have a significant effect on adoption intention.

## 3 Methods

### 3.1 Empirical setting: the adoption of photovoltaic systems

The study is empirically grounded in the adoption of residential photovoltaic (PV) systems through third-party ownership (TPO). A photovoltaic system converts light into electricity using interconnected photovoltaic cells that create a photovoltaic module, a mounting structure for the module, an inverter, a storage battery, and a charge controller [56, p. 4]. PV systems have qualities of prevention regarding emission reduction and climate change prevention [57] as well as lowering dependence on unreliable fossil fuel markets [19].

Ownership of residential solar PV systems can happen either through direct or third-party ownership. Direct ownership occurs when the homeowner owns the equipment and finances the purchase with or without local or national government support. While direct ownership used to be a common choice, buyers have been shifting towards TPO in the last ten years [58]. In TPO, commercial companies own and operate PV systems (either customer-sited or in designated solar parks) and customers can decide whether to lease the PV system or enter a power purchase agreement [58]. TPO can reduce adoption costs, reduce technology risk and complexity, and yield cost savings within the first month, unlike the order of decades of direct ownership [59].

The process to study the adoption of PV systems through TPO started in collaboration with a local electricity company in central Finland in early 2021. The researchers identified the company offers two forms of TPO, which were identified as target behaviors in our study: the rental of a solar panel in a PV park, and the selection of a solar electricity contract. In the first form of TPO, consumers rent a panel located in a solar PV park, and the electricity production of that panel is credited to the electricity bill where the average credit in central Finland is 1 €/month. In the second form of TPO, consumers choose their electricity to be fully procured from solar panel production; specifically, consumers pay a basic monthly fee (approx. 4 €/month in Finland) and a consumption fee (fixed rate per kWh).

### 3.2 Survey design

The survey was designed following the survey designs of Wolske et al. [7] and Elmustapha et al. [47] to capture intentions regarding target behaviors (Appendix A). The first part of the questionnaire included sociodemographic questions including age, sex, and annual household income. This section also assessed the size and type of the house the respondent lives in (detached, semi-detached, apartment block) and the type of management of the housing, meaning if it is owner-occupied, or some form of tenancy. DOI constructs were measured with two to three items, based on previous studies on the adoption of PV systems e.g., Refs. [7,8,12,47,[60], [61], [62]]. Items included in the prevention construct were designed based on the literature on the preventive quality of PV systems regarding emission reduction and climate change prevention e.g. Refs. [56,63]. Answer options were in the form of multiple-choice questions (for gathering sociodemographic data) and in a 5-point Likert scale for remaining variables where 1 reflected strong disagreement and 5 reflected strong agreement.

The survey was piloted to ten respondents before its official distribution. Pilot respondents were selected by the research team seeking to include individuals that represented diverse age groups, housing types, and educational backgrounds. After piloting a few questions were modified (rewording to simplify questions) and the overall evaluation of the survey was positive.

### 3.3 Survey distribution, and responses

The survey was distributed through the website of the electricity company in central Finland from September to November 2021. This sampling method represents convenience, non-probability sampling [64]; even though the survey is open to all respondents, the distribution channel introduces a self-selection bias. To alleviate this bias, we collected demographic data which was then compared to the general population. Out of the total number of responses (N = 365), the researchers eliminated 81 responses that were not filled out completely, yielding a final set of N = 284. This sample size fulfills the minimum sample size for a Partial Least Squares model where the sample should be ten times the largest number of formative indicators measuring one construct [65].

For sociodemographic variables, there were respondents who “Prefer not to say” their sex (2.1 %) and income (13.4 %) and this is not considered to create reporting bias. Table 1 reports participants' sociodemographic information. When comparing our sample with statistics of the Finnish population [66], this sample represents certain characteristics more than others. Sex and household income were close representations of national averages. While respondent age was mostly distributed similarly to the country's age distribution, respondents under 25 were overrepresented with 24 % in our sample (making up 11 % of the population in Finland) and respondents over 65 were underrepresented with no responses in our sample (making up 23 % of the population).

**Table 1:** Participant information.

Sex	n	Percentage (%)	National average (%)
Male	108	38.1	49.5
Female	170	59.8	50.5
Prefer not to say	6	2.1	
**Age group**			
18–25	68	23.9	11
25–35	42	14.8	13
36–45	50	17.6	13
46–55	62	21.8	12
56–65	62	21.8	13
Over 65	0	0	23
**Type of household**			
Detached house	81	28.5	38
Semi-detached house	31	10.9	13
Apartment block	156	54.9	47
Other	16	5.7	2
**Household income**			
Under € 9999	7	2.5	The mean household income in Finland is ∼33,000 EUR/year [66]
€ 10,000 - € 19,999	38	13.4	
€ 20,000- € 39,999	82	28.9	
€ 40,000 - € 69,999	60	21.1	
€ 70,000–99,999	37	13	
€ 100,000–150,000	18	6.3	
More than 150,000 €	4	1.4	
I don't want/can't say	38	13.4	
N	284	100	

### 3.4 Data analysis: PLS-SEM

This study sought to conceptualize the preventive quality as a construct of innovations and identify how the preventive quality of innovations influences adoption intention, for which it relied on Partial Least Squares Structural Equation Modelling, PLS-SEM [67] as a data analysis method. PLS-SEM seeks to confirm theories by determining how well a model can estimate a sample data matrix [68]. PLS-SEM is most useful when the analysis seeks to test a theoretical framework from a prediction perspective and when research is exploratory, as in our setting [68]. Before data analysis, survey items that were negatively phrased (REL3R and CPLXR) were reversed-coded in SPSS to ensure item consistency. Following Hair et al. [65], Partial Least Square-Structural Equation Modeling (PLS-SEM) was implemented using the software SmartPLS4.

### 3.5 Initial model specification

The structural model, or inner model, displays relationships between the constructs under evaluation. The measurement model, or the outer model, evaluates the relationship between indicator variables and their corresponding constructs [65]. The exogenous model is reflective, per Coltman et al.’s guidelines [69]. In a reflective model, the latent construct exists independent of the measures; these models assume that causality flows from construct to indicators and indicators share a theme with high intercorrelations. Reflective models are the most common models in business literature. Moreover, in formative models, the latent construct depends on the interpretation by the scholar, for example, a composite measure made up of several components that cannot exist by itself. Causality is the opposite as in reflective models, where it flows from the indicators to the construct and items do not necessarily share the same theme nor have a pattern of intercorrelation [69]. It is important to identify and highlight the nature of the measurement model to ensure the reliability and validity of research findings [67].

**Fig. 1:**
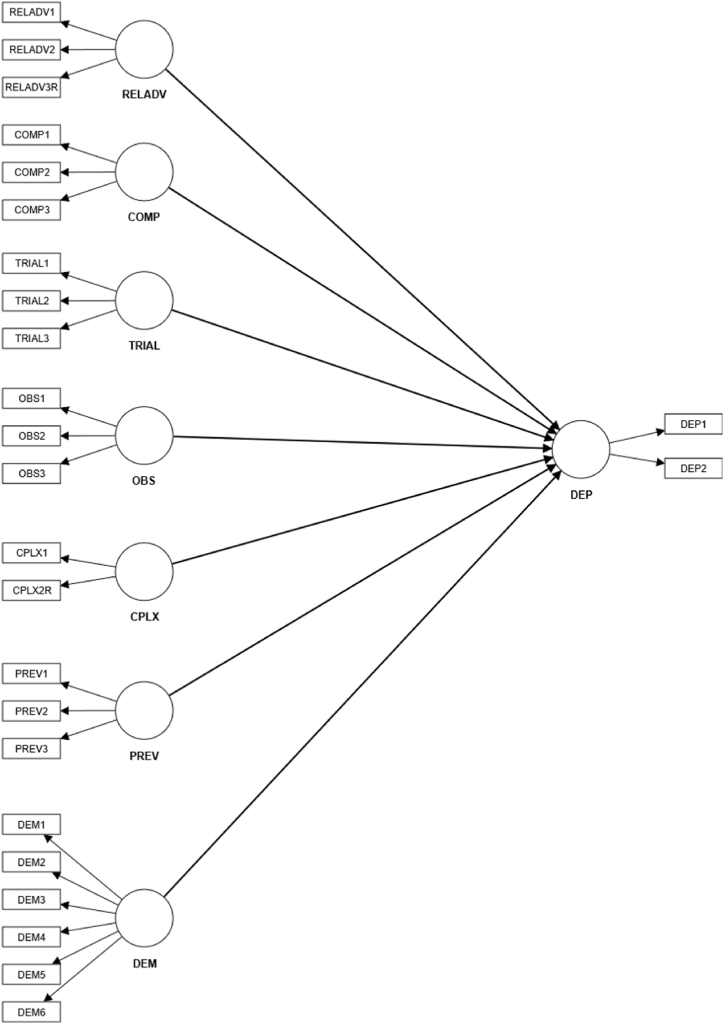
Initial PLS-Model.

The structural model (Fig. 1) shows the relationships between latent exogenous and endogenous variables [65]. Exogenous constructs act as independent variables; these are based on innovation attributes as presented in Rogers [42], and the preventive quality of PV systems about emission reduction and climate change prevention [63]. The endogenous construct acts as the dependent variable, and it is explained by other constructs. The two items in this construct were designed by the four researchers involved in creating the survey to investigate the adoption intention of local TPO PV systems; the design of these items was adapted from previous studies measuring the adoption intention of PV systems [70]. As both adoption models (rental of a panel and solar electricity contracts) belong to TPO and possess overlapping characteristics — no upfront investment, reduced complexity, and low technology risk [55] — they were incorporated into a single construct representing adoption intention.

## 4 Data analysis and results

### 4.1 Common method bias

To assess common method bias, Harman's single factor test was conducted in SPSS. The test revealed that the total variance extracted by one factor is 28.23 % which is below the recommended threshold value of 50 %. Therefore, we find that the common method bias is not an issue in our study.

### 4.2 Reliability and validity

To assess the reliability and validity of the measurement model (Table 2), we ran the PLS-SEM algorithm and bootstrapping technique to test the structural model's accuracy [65]. As the measurement model is made up of reflectively measured constructs [69] reliability and validity were assessed using evaluative measures fit for reflective models. First, item reliability was measured through indicator loadings (λ) [68], in this step, items with values below 0.5 were removed, where four variables in total were removed (DEM1-3, TRIAL1).

**Table 2:** Reliability and validity assessment.

Item	λ	ρA	AVE	VIF
**Dependent variable**		0.765	0.783	
DEP1	0.919			1.487
DEP2	0.849			1.487
**Demographics**		1.456	0.634	
DEM4	0.973			1.953
DEM5	−0.823			1.892
DEM6	0.529			1.318
**Prevention**		0.799	0.714	
PREV1	0.816			1.562
PREV2	0.875			2.094
PREV3	0.842			1.766
**Relative advantage**		0.761	0.623	
REL1	.831			1.788
REL2	.903			1.878
REL3R	.603			1.134
**Trialability**		0.750	0.800	
TRIAL2	0.894			1.564
TRIAL3	0.895			1.564
**Observability**		0.622	0.539	
OBS1	0.584			1.168
OBS2	0.783			1.375
OBS3	0.813			1.205
**Compatibility**		0.834	0.719	
COMP1	0.887			1.813
COMP2	0.822			1.724
COMP3	0.834			1.724
**Complexity**		1.072	0.748	
CPLX1	0.759			1.424
CPLX2R	0.960			1.424

Second, to assess the construct's internal consistency reliability, Rho_A (ρA) was selected following more recent guidelines [71] that identify ρA as a consistent reliability coefficient for PLS [67]. Following Hair et al. [68], all values are within acceptable levels of a minimum of 0.60.

Third, each construct's validity was measured with the average variance extracted (AVE) where all values were above 0.5 [68]. Fourth, discriminant validity was assessed with the Heterotrait-monotrait (HTMT) ratio [72] with a cutoff value of 0.90. Here, the HTMT between relative advantage and observability was above 1; however, an analysis of the Fornell & Larcker criterion [73] (Table 3) confirms there is no validity issue between these two items.

**Table 3:** Fornell & Larcker criterion.

	COMP	CPLX	DEM	DEP	OBS	PREV	RELADV	TRIAL
COMP	**0.848**							
CPLX	0.169	**0.865**						
DEM	0.093	−0.004	**0.796**					
DEP	0.361	0.109	0.072	**0.885**				
OBS	0.552	0.06	0.043	0.343	**0.734**			
PREV	0.627	0.052	0.167	0.404	0.489	**0.845**		
RELADV	0.563	0.101	0.089	0.397	0.678	0.595	**0.789**	
TRIAL	0.094	0.371	−0.108	0.127	0.269	0.018	0.096	**0.895**

**Fig. 2:**
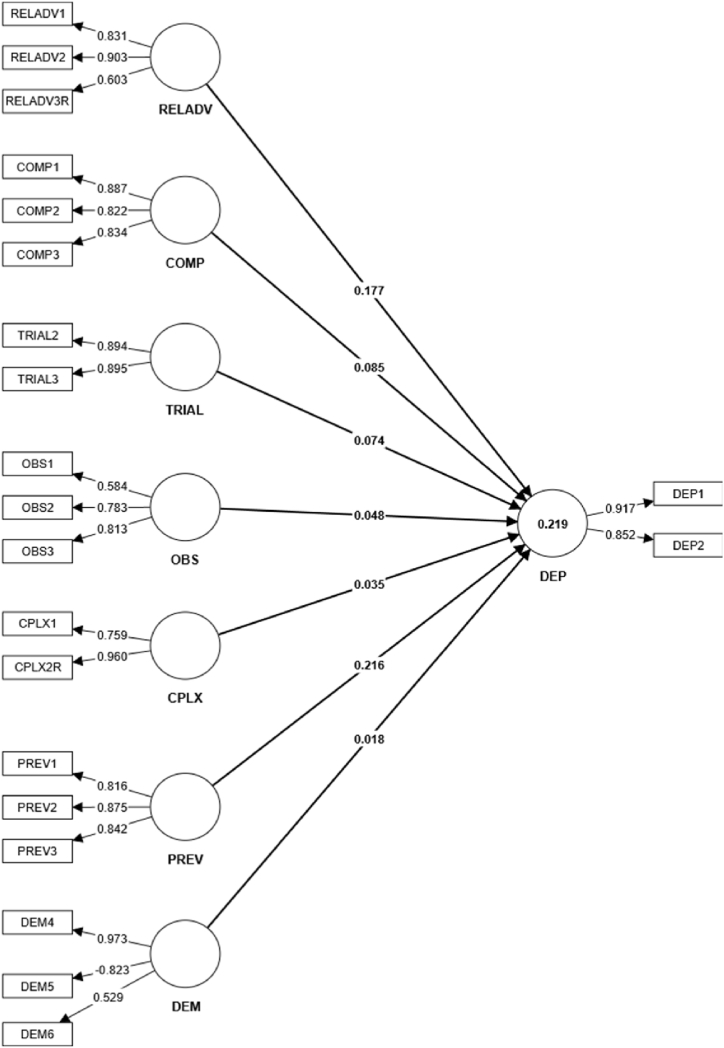
Modified PLS-SEM -model.

We then evaluated the reliability and validity of the structural model. We first assessed multi-collinearity with the value of each indicator's Variance Inflation Factor (VIF) and identified no issues as all values fell below 3. Furthermore, we relied on the coefficient of determination (R^2^) and path coefficients to assess the model's ability to predict the endogenous constructs where R^2^ and R^2^-adj values were 0.21 and 0.20 respectively. Based on results from the reliability and validity assessment, four items were removed from the model due to very low loadings.^1^ The modified PLS model with outer loadings, total effects, and R^2^ is shown in Fig. 2.

### 4.3 Outer loadings

Outer loadings and T-statistics from the bootstrapping procedure^2^ of each group are presented in Table 4. The analysis shows all indicators are highly significant (p < 0.01) except for demographic items. The absolute contribution of each item to the latent variable is shown in Fig. 2 (Modified PLS).

**Table 4:** Significance of outer loadings of the modified PLS-SEM Model.

	Original sample	Sample mean	Standard deviation	T statistics	P values
COMP1 <- COMP	0.887	0.888	0.019	46.111	0.000
COMP2 <- COMP	0.822	0.819	0.034	24.157	0.000
COMP3 <- COMP	0.834	0.832	0.032	26.225	0.000
CPLX1 <- CPLX	0.758	0.699	0.256	2.962	0.003
CPLX2R < - CPLX	0.960	0.885	0.219	4.388	0.000
DEM4 <- DEM	0.973	0.504	0.659	1.477	0.140
DEM5 <- DEM	−0.822	−0.356	0.663	1.240	0.215
DEM6 <- DEM	0.530	0.385	0.489	1.084	0.278
DEP1 <- DEP	0.849	0.851	0.027	31.129	0.000
DEP2 <- DEP	0.583	0.574	0.102	5.718	0.000
OBS1 <- OBS	0.783	0.775	0.050	15.670	0.000
OBS2 <- OBS	0.813	0.813	0.049	16.728	0.000
OBS3 <- OBS	0.816	0.817	0.029	27.752	0.000
PREV1 <- PREV	0.875	0.874	0.019	46.534	0.000
PREV2 <- PREV	0.842	0.841	0.027	31.441	0.000
PREV3 <- PREV	0.830	0.829	0.037	22.479	0.000
RELADV1 <- RELADV	0.903	0.901	0.022	40.699	0.000
RELADV2 <- RELADV	0.603	0.599	0.076	7.958	0.000
RELADV3R < - RELADV	0.894	0.871	0.138	6.489	0.000
TRIAL2 <- TRIAL	0.896	0.872	0.133	6.729	0.000
TRIAL3 <- TRIAL	0.919	0.916	0.014	64.422	0.000

*Two-tailed *t*-test. Critical t-value for significance level is 1.96 for 5 %.

** Statistical significance if p-value<0.05.

### 4.4 The predictive power of the model

The predictive power of a PLS model requires non-parametrical tests that evaluate its quality; criteria include R^2^, T-values, and path coefficients [65]. The coefficient of determination of the structural model (R^2^ = 0.219, SD = 0.038) represents small yet acceptable levels of predictive accuracy. Other measures (p-value = 0.000, *t = 5*.757) are also well within acceptable levels [68].

### 4.5 Path coefficients of the structural model: hypotheses testing

Table 5 shows the path coefficients of the structural model and their corresponding hypotheses. These results signal that the constructs of prevention and relative advantage affect intentions to adopt TPO PV systems where the strongest effect comes from prevention items based on p-values and T-statistics. Other constructs do not predict respondents’ intentions to adopt TPO PV systems.

**Table 5:** Path coefficients of the structural model.

	Original sample	Sample mean	Standard deviation	T statistics^a^	P values^b^
H1: PREV - > DEP	0.217	0.212	0.073	**2.980**	**0.003**
H2: RELADV - > DEP	0.177	0.179	0.080	**2.199**	**0.028**
H3: TRIAL - > DEP	0.074	0.078	0.062	1.205	0.228
H4: OBS - > DEP	0.047	0.054	0.084	0.565	0.572
H5: COMP - > DEP	0.085	0.086	0.063	1.351	0.177
H6: CPLX - > DEP	0.035	0.040	0.070	0.503	0.615
H7: DEM - > DEP	0.017	0.029	0.075	0.230	0.818

PREV= Preventive quality; RELADV= Relative advantage; TRIAL = Trialability.

OBS= Observability, COMP= Compatibility, CPLX= Complexity, DEM = Demographic information, DEP = Dependent variable.

a Two-tailed *t*-test. Critical t-value for significance level is 1.96 for 5 %.

b Statistical significance if p-value<0.05.

## 5 Discussion and conclusions

The objective of this study was to conceptualize the preventive quality as a construct of innovations and identify how the preventive quality of innovations influences adoption intention. Based on previous empirical settings, we hypothesized respondents would exhibit a greater intention to adopt from perceptions of relative advantage, trialability, observability, and compatibility of the innovation. We also hypothesized respondents would exhibit a negative intention to adopt based on the complexity and preventive quality of innovations. Our findings indicate that the preventive quality of innovations can be a standalone construct of innovations, and it has a significant and positive impact on adoption intention, yielding a greater impact than all other studied constructs, thus addressing our research objectives.

As for our hypothesized relationships, we found that all the constructs under study, attributes of innovations (relative advantage, trialability, observability, compatibility, and complexity), the preventive quality, and demographic factors, have a positive influence over adoption intention. We identified no constructs with a negative influence over adoption intention. However, only two of these constructs had a statistically significant influence over adoption intention: the preventive quality, and relative advantage and help predict intention to adopt TPO PV systems.

While the relative advantage is traditionally considered the most important predictor of the adoption of innovations [42], we found the preventive quality of TPO PV systems to have higher predictive power; this could be explained by the increasing pressure individuals experience regarding sustainability objectives through their actions [74]. The prevalence of prevention in the Finnish context can be explained by the proficient average knowledge about climate change where most Finns believe that climate change is caused by increased carbon dioxide in the atmosphere [75,76]. Moreover, Finns have high environmental awareness and structural skills for making a more sustainable future [76]. Our findings regarding the preventive quality of innovations are in line with previous studies that identify Finnish people as willing to change their consumption habits for the benefit of climate change [77].

While the focus of this study was PV systems, the preventive quality of innovations is inherently present in green and sustainable innovations as green products can perform better in materials, energy, and/or pollution in comparison to conventional products [5]. Thus, our study builds up a fruitful avenue for future research to investigate the adoption process of green innovations.

Understanding the influence of the preventive quality of innovations represents a useful finding for policymakers as it highlights insights to promote the adoption of innovations that create preventive capacity, helping build resilient societies [29]. Particularly, policymakers could promote a favorable evaluation of prevention among potential adopters. While ‘prevention’ might not appeal to all buyers, as economic incentives usually have a stronger influence over other factors [78], benefits incurred from prevention could also be quantified in the form of time and money savings. Moreover, understanding the role of prevention can help appeal to buyers who are keen on preventing an unwanted event (potentially early adopters); more buyers could then follow due to peer influence. Generally, early adopters are less motivated by economic incentives and are driven by other context-specific features, such as environmental concerns in the case of PV systems [54].

Findings provide a step toward building the construct of prevention with the intent to adopt innovations. The main theoretical contribution of our study is that we show that preventive quality has significant and positive explanatory power when explaining adoption intentions, while previous research points to a negative explanatory power over adoption and adoption intention [30,31]. This could be explained by the fact that the perception of benefits related to climate change mitigation is immediate upon TPO-PV system adoption (users know they will now start using renewable electricity) as well as by the characteristics of the Finnish context.

A limitation of this study is the geographic distribution of respondents, all within central Finland, for which results cannot be generalized across national contexts. Furthermore, the over- and under-representation of age groups across respondents limits the generalizability of results within Finland. We suggest that future research should consider expanding the setting to other countries. Furthermore, we focused on a few factors influencing adoption, namely DOI characteristics and demographic factors, and did not include other control variables. This focus limits identifying other elements that influence the adoption as those highlighted in other behavioral theories such as the Technology Acceptance Model, TAM [23, p. 19], the Theory of Planned Behavior, TPB [24], or the Unified Theory of Acceptance and Use of Technology, UTAUT [25]. However, findings from our exploratory study pave the way to incorporate these behavioral theories in future studies. We also suggest studying the role of the preventive quality of other innovations that also display prevention qualities. Future studies should also address the potential interrelationships or mediating effects of prevention over other constructs.

## Data availability statement

Data will be made available on request.

## CRediT authorship contribution statement

**Deborah Kuperstein-Blasco:** Conceptualization, Formal analysis, Writing – original draft, Writing – review & editing. **Saku Mäkinen:** Resources, Supervision.

## Declaration of competing interest

The authors declare that they have no known competing financial interests or personal relationships that could have appeared to influence the work reported in this paper.

## Figures and Tables

**Table A.1:** Survey questions

Measures	Survey questions	Scale	Codes
Adoption intention (dependent variables)	Would you be willing to sign up for a solar contract?	1. Yes, and I would be willing to pay more for electricity. 2. Yes, if it does not affect the price of electricity. 3. Yes, if it reduces the price of electricity. 4. I am not interested	DEP1
	In which period would you be willing to take up a solar contract? [70]		DEP2
Sociodemographic variables	What is your sex?	Male/Female/Don't want to say	DEM1
	Please specify your age group:	18-25/26–35/36-45/46–55/56-65/over 65	DEM2
	What is the total gross income (pre-tax income) of your household per year?	Under 9999€/10,000€ - 19,999€/20,000€- 39,999€/40,000€ - 69,999€/70,000€-99,999€/100,000€-150,000€/More than 150,000€. I don't want/can't say	DEM3
	What is the type of house you live in?	Detached house/Semi-detached or terraced house/Apartment block/Other	DEM4
	What is the floor area of your housing?	Open-ended	DEM5
	What is the type of management of your housing?	Owner-occupied accommodation/Rented accommodation (includes tenanted accommodation)/Tenancy right or shared ownership/Other (includes sub-tenanted housing)	DEM6
Relative advantage	Switching to solar electricity will make a good impression on other people [60]	1. Strongly disagree 2. Somewhat disagree 3. Neither agree nor disagree 4. Somewhat agree 5. Strongly agree	REL1
	Switching to solar electricity will make me feel good [60]		REL2
	I think solar electricity is expensive [7] (Reversed)		REL3R
Compatibility	I welcome solar energy [8]	1. Strongly disagree 2. Somewhat disagree 3. Neither agree nor disagree 4. Somewhat agree 5. Strongly agree	COMP1
	Solar energy is a reliable way to generate electricity [8]		COMP2
	Solar energy as a way of generating electricity is a new and advanced technology [8]		COMP3
Trialability	Before changing my electricity contract, I would like to talk to someone who has already switched to solar electricity [7]	1. Strongly disagree 2. Somewhat disagree 3. Neither agree nor disagree 4. Somewhat agree 5. Strongly agree	TRIAL1
	I have enough information about the solar electricity contract to decide to switch [61]		TRIAL2
	I know where I can get reliable information about solar electricity [61]		TRIAL3
Observability	I know more than one person who has switched their electricity to solar electricity [7]	1. Strongly disagree 2. Somewhat disagree 3. Neither agree nor disagree 4. Somewhat agree 5. Strongly agree	OBS1
	Many people who are important to me think it would be good if I switched my electricity contract to solar electricity [12]		OBS2
	A photovoltaic contract allows me to increase local energy production [62]		OBS3
Complexity	I find it easy to switch to solar electricity [61]	1. Strongly disagree 2. Somewhat disagree 3. Neither agree nor disagree 4. Somewhat agree 5. Strongly agree	CPLX1
	I think it takes a long time to tender solar electricity [7] (Reversed)		CPLX2R
Prevention	I consider it important that switching to photovoltaics allows me to save natural resources and reduce greenhouse gas emissions [62,63]	1. Strongly disagree 2. Somewhat disagree 3. Neither agree nor disagree 4. Somewhat agree 5. Strongly agree	PREV1
	People like me should do all they can to reduce emissions and prevent climate change [47,63]		PREV2
	It is important to use renewable energy to reduce emissions [56,63]		PREV3

**Table B.1:** Initial model's loadings, reliability, and validity assessment

Item	λ	ρA	AVE	VIF
**Dependent variable**		0.772	0.783	
DEP1	0.919			1.487
DEP2	0.849			1.487
**Demographics**		1.464	0.634	
DEM1	0.037			1.152
DEM2	0.558			1.083
DEM3	0.491			1.077
DEM4	−0.670			2.015
DEM5	0.621			1.931
DEM6	0.527			1.386
**Relative advantage**		0.761	0.623	
REL1	.830			1.788
REL2	.903			1.878
REL3R	.603			1.134
**Compatibility**		0.834	0.719	
COMP1	0.887			1.813
COMP2	0.822			1.724
COMP3	0.834			1.724
**Trialability**		0.750	0.800	
TRIAL1	−0.214			1.042
TRIAL2	0.894			1.564
TRIAL3	0.895			1.564
**Observability**		0.622	0.539	
OBS1	0.584			1.168
OBS2	0.783			1.375
OBS3	0.813			1.205
**Complexity**		1.072	0.748	
CPLX1	0.759			1.424
CPLX2R	0.960			1.424
**Prevention**		0.799	0.714	
PREV1	0.816			1.562
PREV2	0.875			2.094
PREV3	0.842			1.766

## Footnotes

1 Detailed results of the reliability and validity tests for the initial model are shown in Appendix B.

2 Complete bootstrap, 5000 subsamples, two-tailed with 0.05 significance.
