# Supplementary material for: Recognizing the preventive quality in the adoption of innovations: The case of third-party ownership photovoltaic systems in Finland
Source: Heliyon. 2023 Nov 12;9(11):e21907. doi: 10.1016/j.heliyon.2023.e21907 (PMC10694153; doi:10.1016/j.heliyon.2023.e21907)
Supplement: Supplementary file 1 [file mmc1.docx]

Survey questions (full questionnaire, same as Appendix A except for the construct of values).

| Measures | **Survey questions** | **Scale** | **Codes** |
| --- | --- | --- | --- |
| Adoption intention (dependent variables) | Would you be willing to sign up for a solar contract? | 1. Yes, and I would be willing to pay more for electricity. 2. Yes, if it does not affect the price of electricity. 3. Yes, if it reduces the price of electricity. 4. I am not interested | DEP1 |
|  | In which period would you be willing to take up a solar contract? |  | DEP2 |
| Sociodemographic variables | What is your sex? | Male/ Female/ Don’t want to say | DEM1 |
|  | Please specify your age group: | 18-25/26-35/36-45/46-55/56-65/over 65 | DEM2 |
|  | What is the total gross income (pre-tax income) of your household per year? | Under 9,999€/10,000€ - 19,999€/ 20,000€- 39,999€/ 40,000€ - 69,999€ /70,000€-99,999€ /100,000€-150,000€ /More than 150,000€. I don't want / can't say | DEM3 |
|  | What is the type of house you live in? | Detached house/ Semi-detached or terraced house/Apartment block/ Other | DEM4 |
|  | What is the floor area of your housing? | Open-ended | DEM5 |
|  | What is the type of management of your housing? | Owner-occupied accommodation/ Rented accommodation (includes tenanted accommodation)/ Tenancy right or shared ownership/ Other (includes sub-tenanted housing) | DEM6 |
| Relative advantage | Switching to solar electricity will make a good impression on other people. | 1. Strongly disagree 2. Somewhat disagree 3. Neither agree nor disagree 4. Somewhat agree 5. Strongly agree | REL1 |
|  | Switching to solar electricity will make me feel good |  | REL2 |
|  | I think solar electricity is expensive (Reversed) |  | REL3R |
| Compatibility | I welcome solar energy | 1. Strongly disagree 2. Somewhat disagree 3. Neither agree nor disagree 4. Somewhat agree 5. Strongly agree | COMP1 |
|  | Solar energy is a reliable way to generate electricity |  | COMP2 |
|  | Solar energy as a way of generating electricity is a new and advanced technology |  | COMP3 |
| Trialability | Before changing my electricity contract, I would like to talk to someone who has already switched to solar electricity | 1. Strongly disagree 2. Somewhat disagree 3. Neither agree nor disagree 4. Somewhat agree 5. Strongly agree | TRIAL1 |
|  | I have enough information about the solar electricity contract to decide to switch |  | TRIAL2 |
|  | I know where I can get reliable information about solar electricity. |  | TRIAL3 |
| Observability | I know more than one person who has switched their electricity to solar electricity | 1. Strongly disagree 2. Somewhat disagree 3. Neither agree nor disagree 4. Somewhat agree 5. Strongly agree | OBS1 |
|  | Many people who are important to me think it would be good if I switched my electricity contract to solar electricity |  | OBS2 |
|  | A photovoltaic contract allows me to increase local energy production |  | OBS3 |
| Complexity | I find it easy to switch to solar electricity | 1. Strongly disagree 2. Somewhat disagree 3. Neither agree nor disagree 4. Somewhat agree 5. Strongly agree | CPLX1 |
|  | I think it takes a long time to tender solar electricity (Reversed) |  | CPLX2R |
| Prevention | I consider it important that switching to photovoltaics allows me to save natural resources and reduce greenhouse gas emissions | 1. Strongly disagree 2. Somewhat disagree 3. Neither agree nor disagree 4. Somewhat agree 5. Strongly agree | PREV1 |
|  | People like me should do all they can to reduce emissions and prevent climate change |  | PREV2 |
|  | It is important to use renewable energy to reduce emissions |  | PREV3 |
| Values | It is important to me to protect nature | 1. Strongly disagree 2. Somewhat disagree 3. Neither agree nor disagree 4. Somewhat agree 5. Strongly agree | VAL1 |
|  | It is important to me that all people have equal opportunities to pursue happiness |  | VAL2 |
|  | It is important to me to do things that I enjoy |  | VAL3 |
|  | High wealth is important to me |  | VAL4 |
